# The Geriatric Surgery: The Importance of Frailty Identification Beyond Chronological Age

**DOI:** 10.3390/geriatrics5010012

**Published:** 2020-02-28

**Authors:** Virginia Boccardi, Luigi Marano

**Affiliations:** 1 Institute of Gerontology and Geriatrics, Department of Medicine, University of Perugia, Santa Maria della Misericordia Hospital, Piazzale Gambuli 1, 06132 Perugia, Italy; 2 Department of Medicine, Surgery and Neurosciences, Unit of General Surgery and Surgical Oncology, University of Siena, 53100 Siena, Italy; luigi.marano@unisi.it

**Keywords:** ageing, challenge, surgery, frailty

In the last fifty years, there has been a great improvement in social and health conditions. This have led to a significant increase in human lifespan as never seen before. In Italy, older persons are the fastest growing sector of society, mainly due to scientific progress but also to a decrease in the natality rate. According to the latest WHO data published in 2018, life expectancy in Italy is 80.5 for males and 84.9 for females, with a total life expectancy of 82.9 [1]. However, ageing has also promoted a progressive high prevalence of chronic age-related conditions, polypharmacy as well life lived in disability [2]. This demographic change has influenced the health care system in ways such as the increase in hospital use. Inevitably, conditions that need surgery are also increasing in an exponential manner. This has progressively changed the definition of the older subject for the surgeons, where a growing number of complex operations are being performed to patients even over 85 years of age. It is well recognized that as people age, surgery and anesthesia can cause greater stress in their bodies’ functions, the recovery may take longer and the complication rate may be higher, representing an important challenge in this field. Frequently, age-related changes in organs, tissues and systems as a whole lead to the loss of functional and cognitive reserve, which may only become clear under a stressful condition, such as surgery. Often, deciding if a patient is too old for surgery is still problematic, and increasing age itself may represent an important risk factor for operative and postoperative morbidity and mortality [3]. In 1993, Dr Lubin tried with an excellent article to respond the question "ls age a risk factor for surgery?”. The answer was very concise and short—“yes, and no, depending on how you look at the data” [4]. Looking only at the raw statistics, there is a definite and significant increase in mortality along with aging, but when correcting for other factors such as physiologic changes, comorbidities, the types of surgery and the timing of surgical intervention, the results change. Recent studies have clearly shown that age itself is not a prognostic risk factor for complications after elective surgery in older patients, whereas cognitive or functional frailty is [5]. This could be the problem, because frailty—or the state of physical and cognitive vulnerability and the lack of resilience to stressors—is often misunderstood, not identified and often confused as a hallmark of aging. Thus, it is not rare that among cancer patients, the likelihood of being referred for surgery was lower for older people, despite clinical evidence that post-operative recovery outcomes are not dependent on age. Neither a referring physician nor an assessing surgeon should deny patients surgery purely based on chronological age. Instead, decisions should be based on a CGA (comprehensive geriatric assessment) with a precise picture of the patient taking into account the cognitive, functional, nutritional, socioeconomic and affective status [6]. Where surgeons have looked beyond age to recognize the importance of co-morbidities and physiological derangement, they have repeatedly demonstrated that selected patients can have good outcomes. The early multidisciplinary management of older patients can really influence outcome. The surgical risk should be calculated after a valuation of the person beyond his or her birthdate. In 2011, Ellis et al. published a Cochrane review of the use of CGA in old age patients admitted to hospital [7]. The review identified significant reduction in dependence and mortality at one year in patients receiving CGA as compared with a group on traditional assessment. However, despite good evidence in acute medical patients, the surgical community has been slow to adopt this collaborative model of care in older patients. In the elective surgery setting, Harari and colleagues pioneered work examining the role and effectiveness of multidisciplinary geriatric involvement in the care of old age patients undergoing elective surgery. They showed impressive improvements in morbidity and mortality [8]. A recent systematic review examining the use of preparative CGA in surgical patients identified five studies showing encouraging results on postoperative outcomes in very old patients. The most compelling current evidence comes from the orthopedic community, which has embraced the idea of multidisciplinary team (MDT) care and orthogeriatricians. There is evidence that the involvement of an orthogeriatrician can lead to reduced length of stay, reduced mortality, better teamwork, and improved discharge planning. Even high-risk patients may find elective surgery, for colorectal cancer for instance, preferable to emergency treatment for complications such as a bowel obstruction. It is from the team-based discussion of such results that the most proper treatment can be tailored, surgery invasiveness and duration critically analyzed and, if needed, modified, and the best perioperative strategy carefully tailored. However, still few centers provide geriatric medicine advice regarding the care of older surgical patients [9]. Preoperative evaluation, postoperative care, pain control, nutritional support, delirium prevention, mobilization, and rehabilitation are necessary to promote surgery in the elderly and reduce mortality and health care system costs. Surgeons cannot make this change alone but the presence of an expert MDT, including geriatricians, anesthetists, critical care specialists, specialist nurses, therapists, and dieticians, seems to be mandatory in the modern aged world. 
